# The Effects of Cognitive Training on Brain Network Activity and Connectivity in Aging and Neurodegenerative Diseases: a Systematic Review

**DOI:** 10.1007/s11065-020-09440-w

**Published:** 2020-06-12

**Authors:** Tim D. van Balkom, Odile A. van den Heuvel, Henk W. Berendse, Ysbrand D. van der Werf, Chris Vriend

**Affiliations:** 1 Amsterdam UMC, Psychiatry, Amsterdam Neuroscience, Vrije Universiteit Amsterdam, De Boelelaan, 1117 Amsterdam, Netherlands; 2 Amsterdam UMC, Anatomy and Neurosciences, Amsterdam Neuroscience, Vrije Universiteit Amsterdam, De Boelelaan, 1117 Amsterdam, Netherlands; 3 Amsterdam UMC, Neurology, Amsterdam Neuroscience, Vrije Universiteit Amsterdam, De Boelelaan, 1117 Amsterdam, Netherlands

**Keywords:** Cognitive training, Neuroimaging, Magnetic resonance imaging, Network, Neurodegenerative diseases, Aging

## Abstract

Cognitive training (CT) is an increasingly popular, non-pharmacological intervention for improving cognitive functioning in neurodegenerative diseases and healthy aging. Although meta-analyses support the efficacy of CT in improving cognitive functioning, the neural mechanisms underlying the effects of CT are still unclear. We performed a systematic review of literature in the PubMed, Embase and PsycINFO databases on controlled CT trials (*N* > 20) in aging and neurodegenerative diseases with pre- and post-training functional MRI outcomes up to November 23rd 2018 (PROSPERO registration number CRD42019103662). Twenty articles were eligible for our systematic review. We distinguished between multi-domain and single-domain CT. CT induced both increases and decreases in task-related functional activation, possibly indicative of an inverted *U*-shaped curve association between regional brain activity and task performance. Functional connectivity within ‘cognitive’ brain networks was consistently reported to increase after CT while a minority of studies additionally reported increased segregation of frontoparietal and default mode brain networks. Although we acknowledge the large heterogeneity in type of CT, imaging methodology, in-scanner task paradigm and analysis methods between studies, we propose a working model of the effects of CT on brain activity and connectivity in the context of current knowledge on compensatory mechanisms that are associated with aging and neurodegenerative diseases.

**Electronic supplementary material:**

The online version of this article (10.1007/s11065-020-09440-w) contains supplementary material, which is available to authorized users.

## Introduction

In recent years, cognitive training (CT) has become increasingly popular as a treatment for cognitive dysfunction and decline. CT is a behavioral, non-pharmacological treatment that has a history of more than a century but has regained interest in the past two decades (Katz, Shah, & Meyer, 2018). Its scope nowadays ranges from alleviating cognitive dysfunction in neurodegenerative diseases, in which pharmacological treatment options have limited efficacy (Tan et al., 2014; Seppi et al., 2011), to improvement of cognitive abilities in cognitively intact individuals (see e.g., Shah, Weinborn, Verdile, Sohrabi, & Martins, 2017). The recent popularity of CT has evoked substantial debate and criticism from the scientific community regarding its efficacy and validity (van Heugten, Ponds, & Kessels, 2016; Orban, Rapport, Friedman, & Kofler, 2014; Goodier, 2009; Rabipour & Raz, 2012), at least partly due to the growth of commercial companies promising to enhance mental fitness or cure cognitive dysfunction in an aging society by offering online ‘brain training’ products without scientific basis.

Meta-analyses of studies in multiple neurodegenerative diseases support the efficacy of CT to improve cognitive function (Leung et al., 2015; Sitzer, Twamley, & Jeste, 2006; Chandler, Parks, Marsiske, Rotblatt, & Smith, 2016; Dardiotis et al., 2018). Even more so, CT has been shown to delay cognitive decline in both healthy adults and patients with Parkinson’s disease (e.g. Petrelli et al., 2014; Rebok et al., 2014; Willis et al., 2006). This indicates that CT may have a neuroprotective effect that counteracts or delays age- and disease-related degeneration of the brain and is reminiscent of the “use it or lose it” principle (Swaab, 1991; Hultsch, Hertzog, Small, & Dixon, 1999).

The aim of CT through ‘process-based’ or ‘drill’ training is, as generally opposed to cognitive strategy training, to improve cognition through repeated engagement of cognitive processes using one or more challenging and preferably adaptive tasks – analogous to physical training. Stimulation of neuroplasticity and thereby enhancement of cognitive reserve is thought to represent the underlying neurobiological mechanism (Park & Bischof, 2013; Raichlen & Alexander, 2017). Neuroplasticity refers to the brain’s ability to undergo structural and functional alterations by altering neurotransmission, synaptogenesis and neurogenesis from birth to old age (Mahncke, Bronstone, & Merzenich, 2006). Research in rodents has indicated that training-induced neuroplasticity entails changes in dendritic spine formation rate (Lai, Franke, & Gan, 2012), cortical spine density (Kuhlman, O'Connor, Fox, & Svoboda, 2014) and synapse potentiation (Xiong, Znamenskiy, & Zador, 2015; for a review see Abraham, 2008) and also neural changes such as hippocampal synaptic connectivity in animal models of neurodegenerative diseases (Stuart et al., 2017).

In humans, non-invasive neuroimaging techniques are an elegant way to map neural alterations in response to CT, but so far there is no agreement on how CT alters the aging brain at this macroscopic level. This review seeks to provide a systematic overview of reported changes in brain activity and connectivity induced by process-based CT, measured by functional magnetic resonance imaging (MRI). As integration of information in large-scale brain networks is increasingly thought to be essential for successful cognition (Bassett & Sporns, 2017; Bullmore & Sporns, 2009), we aim to describe results in the context of the human brain connectome. This systematic review aims to answer the following questions: what is the influence of the trained domain (e.g., single-domain versus multi-domain, working memory versus attention) on alterations of brain activity and connectivity? Do these alterations specifically involve certain brain networks? Are the mechanisms of effect of CT dependent on the target study population?

Insight into the neural mechanisms of CT across neurodegenerative diseases and healthy elderly, and across training packages is warranted to further optimize the efficacy of CT. We hypothesize that CT-induced changes in brain activity and connectivity are dependent on the type of CT, and that these changes occur predominantly in brain networks important for the specific cognitive domain that is trained. We first summarize the existing literature for multi-domain and single-domain CT separately. In the final section, we evaluate and integrate the reviewed studies, and provide recommendations for future research.

## Methods

### Study Selection and Screening

We performed a systematic literature review following Preferred Reporting Items for Systematic Reviews and Meta-Analyses (PRISMA, http://www.prisma-statement.org/) guidelines on records published in the PubMed, Embase, and PsycINFO databases up to November 23, 2018. We registered the review protocol with the PROSPERO International Prospective Register of Systematic Reviews of the University of York (registration number: CRD42019103662). The search terms were defined as a combination of “cognitive training” and related terms and “neuroimaging” and related terms. Additionally, we added exclusion terms based on our eligibility criteria. An overview of the literature search terms is provided in Electronic Supplementary Material 1. We also added studies from reference lists of studies in our literature search (i.e., snowball method). Records were first independently screened (TvB, CV) for eligibility and disagreement was re-evaluated to consensus.

Eligibility criteria were 1) randomized and non-randomized controlled trials on process-based CT, 2) in a human population of patients suffering from neurodegenerative diseases *or* healthy elderly (defined as a mean age of 60 years or older), 3) with neuroimaging data before and immediately following CT, 4) with functional magnetic resonance imaging (fMRI, i.e., resting-state and/or task-related) as reported outcome measure, and 5) with a minimal total sample size of 20 participants to enhance reliability of single-study results. Eligibility criterion 2 was retrospectively adjusted and therefore deviates from the PROSPERO review protocol: the minimal age for healthy participants was increased from 30 to 60 years in order to include studies specifically in the aging healthy elderly sample. One study that was originally included (Clark, Lawlor-Savage, & Goghari, 2017) was for this reason excluded from the synthesis. Exclusion criteria were combination treatments, such as CT combined with physical activity. As the popularity of process-based CT is increasing, there is also considerable debate on the efficacy and working mechanism, as described in the Introduction. Cognitive strategy training studies were therefore not eligible for this review in order to improve our understanding of –specifically– process-based training, to enhance comparability in a heterogeneous research field.

We only considered reports written in English. Potentially eligible records were screened in full-text based on the aforementioned criteria and excluded records were assigned an exclusion label. Eligible records were included in the systematic review.

While meta-analytic methods such as activation likelihood estimation and seed-based *d* mapping (Eickhoff, Bzdok, Laird, Kurth, & Fox, 2012; Radua et al., 2012) are highly suitable to define agreement across multiple neuroimaging studies, we did not perform such a meta-analysis as the correspondence in study methods, analysis methods, seed regions or regions-of-interest used, and in-scanner tasks was insufficient and did not adhere to recent neuroimaging meta-analysis recommendations (Muller et al., 2018). Additionally, as there already was significant heterogeneity in fMRI study methodology and to ensure comparability of results, we focused on studies using this neuroimaging modality and did not include other modalities (e.g., positron emission tomography, magnetoencephalography) as well.

### Data Extraction and Assessment

We extracted data of participant demographics and intervention characteristics, including sample size, age, domain of CT, control group type (active of passive) and number of hours trained. We classified interventions as multi-domain or single-domain CT. We extracted the specific domain that single-domain interventions focused on (i.e., cognitive control, inhibition, processing speed and working memory). Concerning the fMRI outcome, we extracted the imaging modality (resting-state or task-related fMRI), the analysis method and seed region or region-of-interest (ROI) applied. We extracted the reported regions of CT-related alterations, including directionality of effect, outcome measure, coordinates of the anatomical brain locations (if specified) and correlations with behavioral outcomes (when applicable). Talairach coordinates were converted to Montreal Neurological Institute (MNI) coordinates using Wake Forest University PickAtlas in Statistical Parametric Mapping (Maldjian, Laurienti, & Burdette, 2004; Maldjian, Laurienti, Kraft, & Burdette, 2003). We used BrainNet Viewer to visualize results (Xia, Wang, & He, 2013). Extracted training-induced changes in brain function are relative to a control condition (i.e., the interaction-effect) unless otherwise specified in this paper.

To get an overview of how CT influenced brain network function, we assigned neural network labels to extracted coordinates of neural alterations using the atlases of cerebral, cerebellar and striatal parcellation studies of which the default mode network (DMN), frontoparietal network (FPN), and dorsal and ventral attention network (DAN and VAN) play a large role in facilitating cognitive functions (see Box 1; Power et al., 2011; Fox et al., 2005; Yeo et al., 2011). We used the widely accepted 7-network topology for neocortical areas as described in the Yeo et al. (2011) paper, the Buckner, Krienen, Castellanos, Diaz, and Yeo (2011) paper for cerebellar network organization, the paper by Choi, Yeo, and Buckner (2012) for striatal areas, and classified the hippocampus as a DMN region (Greicius, Krasnow, Reiss, & Menon, 2003; Greicius, Srivastava, Reiss, & Menon, 2004).

**Box 1 Resting-state ‘cognitive’ networks**

**Table:** 

*Default mode network (DMN)* - Essential regions of the DMN are the medial prefrontal cortex and posterior cingulate cortex (PCC). This network is characterized by activity in the absence of external cognitive demand. The DMN is thought to be involved in integration of memory, self-monitoring and theory of mind (Spreng, Mar, & Kim, 2009; Burianova, McIntosh, & Grady, 2010; Jeong, Chung, & Kim, 2015). *Frontoparietal network (FPN) -* Important FPN regions are the dorsolateral prefrontal cortex (dlPFC) and the posterior parietal cortex. This network is also often referred to as the central executive network, and its activity is anti-correlated with DMN activity. The FPN has previously been described as a “multi-demand network” (Duncan, 2010), and its activity is important for goal-directed cognitive tasks, including working memory, planning, judgment and decision-making (Kelly, Uddin, Biswal, Castellanos, & Milham, 2008; Menon, 2011). Relative to other brain networks, the FPN shows strong within- as well as between-network connectivity, reflecting the heterogeneous functions it encompasses (Cole et al., 2013). *Dorsal attention network (DAN) & ventral attention network (VAN) -* Lastly, two attention networks can be distinguished that follow a dorsal route (DAN) or a ventral route (VAN). The DAN consists of important regions in the intraparietal sulcus and frontal eye field, and it is mainly important for voluntary, goal-directed attention orientation (Fox, Corbetta, Snyder, Vincent, & Raichle, 2006; Fox et al., 2005; Corbetta & Shulman, 2002; Corbetta, Kincade, Ollinger, McAvoy, & Shulman, 2000). The VAN directs attention stimulus-driven – that is, when identifying salient stimuli (Menon & Uddin, 2010; Sridharan, Levitin, & Menon, 2008; Fox et al., 2005, 2006; Corbetta et al., 2000). The VAN is therefore also referred to as the “salience network” (Seeley et al., 2007; Menon & Uddin, 2010). Crucial VAN regions are the anterior insula and dorsal anterior cingulate cortex.	

### Quality Assessment

We assessed the quality of individual studies by a National Institutes of Health standardized quality assessment tool of controlled intervention studies (https://www.nhlbi.nih.gov/health-topics/study-quality-assessment-tools, see Electronic Supplementary Material 2). Criteria involved clear trial description, randomization procedure, allocation concealment, blinding, no baseline differences between groups, low drop-out rate and difference in drop-out between groups, high protocol adherence, similar simultaneous treatments (e.g., treatment-as-usual), outcome measure quality, sufficient power, pre-specified analyses, and intention-to-treat analysis. The neuroimaging analysis quality was additionally assessed by a clear description of neuroimaging protocol and analyses, correction for motion and multiple comparisons correction. We coded trials as ‘Poor’ if they adhered to <10 criteria, ‘Fair’ if they adhered to 10–13 criteria and ‘Good’ if they adhered to >13 criteria. Records were independently assessed by TvB and CV and disagreement was re-evaluated to consensus. As the final sample size was low, we did not exclude studies from this review based on the quality.

## Results

### Study Selection

**Fig. 1:**
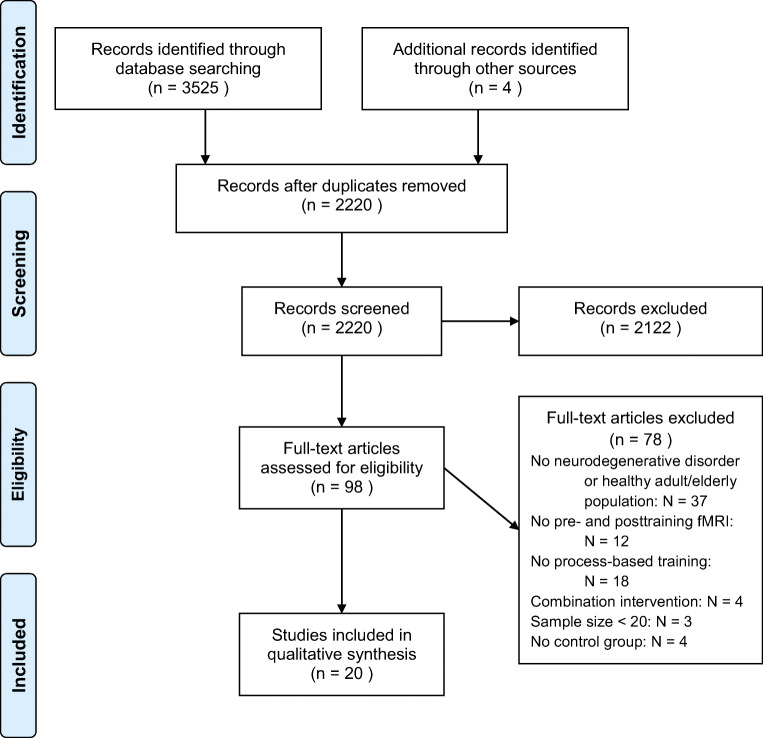
Flow diagram of the screening process according to PRISMA guidelines

The literature screening resulted in 1760 records through PubMed, 1058 records through Embase and 707 records through PsycINFO. Of these, 1300 records were duplicates and five records were non-English. A total of 2220 records were screened of which 98 full-text articles were reviewed. Twenty full-text articles met the inclusion criteria and were included in this systematic review. An overview of the excluded records that underwent full-text review, including the main reason for exclusion, is provided in Electronic Supplementary Material 3. A flow diagram of the screening process according to PRISMA guidelines (www.prisma-statement.org) is provided in Fig. 1.

### Study Characteristics

Eight studies included healthy elderly (Cao et al., 2016; De Marco et al., 2016; Luo et al., 2016; Li et al., 2016; Kim, Chey, & Lee, 2017; Kuhn et al., 2017; Lebedev, Nilsson, & Lovden, 2018; Ross et al., 2018), three studies included individuals with mild cognitive impairment (MCI; Suo et al., 2016; Lin et al., 2016; De Marco, Meneghello, Pilosio, Rigon, & Venneri, 2018), one study was performed in Alzheimer’s disease (AD; Huntley, Hampshire, Bor, Owen, & Howard, 2017), six in multiple sclerosis (MS; De Giglio et al., 2016; Bonavita et al., 2015; Parisi et al., 2014; Filippi et al., 2012; Campbell, Langdon, Cercignani, & Rashid, 2016) and one study was performed in Parkinson’s disease (PD; Diez-Cirarda et al., 2016). One study included AD and MCI patients, and healthy elderly (Barban et al., 2017). The sample size varied from twenty (Filippi et al., 2012; Parisi, Rocca, Valsasina, et al., 2014) to 54 participants (Li et al., 2016). Fourteen studies assessed the neural effects of multi-domain training (Barban et al., 2017; Bonavita et al., 2015; Campbell et al., 2016; Cao et al., 2016; De Giglio et al., 2016; De Marco et al., 2016, 2018; Diez-Cirarda et al., 2016; Filippi et al., 2012; Li et al., 2016; Luo et al., 2016; Parisi, Rocca, Valsasina, et al., 2014; Suo et al., 2016) and six studies assessed the effects of single-domain training (Huntley et al., 2017; Kim et al., 2017; Kuhn et al., 2017; Lebedev et al., 2018; Lin et al., 2016; Ross et al., 2018). A description of the interventions and re-coding to single- or multi-domain training is supplied in Electronic Supplementary Material 4. The amount of training hours varied from ten (Ross et al., 2018) to 78 h (Suo et al., 2016). The majority of studies (i.e., *n* = 13) compared CT effects to an active control group (Barban et al., 2017; Bonavita et al., 2015; Campbell et al., 2016; Cerasa et al., 2013; De Marco et al., 2016, 2018; Diez-Cirarda et al., 2016; Huntley et al., 2017; Lebedev et al., 2018; Li et al., 2016; Lin et al., 2016; Ross et al., 2018; Suo et al., 2016), six studies applied a passive control group (Cao et al., 2016; De Giglio et al., 2016; Filippi et al., 2012; Kim et al., 2017; Luo et al., 2016; Parisi, Rocca, Valsasina, et al., 2014) and one study compared CT to both an active and a passive control group (Kuhn et al., 2017). All studies used randomization except for three studies that were not described to be randomized (Bonavita et al., 2015; De Marco et al., 2016; Kim et al., 2017) and two studies that were unclear on randomization (De Marco et al., 2018; Lin et al., 2016). Detailed study characteristics are shown in Table 1.

**Table 1:** Overview of multi-domain and single-domain cognitive training studies using an fMRI outcome

author	population	N	age (range)*	CT (domain)	CG	length	imaging	task	method	seed/ROI	region	effect	outcome measure
Cao et al., 2016^1^	Healthy elderly	32	69.8 (65–75)	MD	passive	24 one-hour group sessions, twice a week	rs-fMRI		seed	PCC	PCC - dlPFC L	–	FC
											PCC - cerebellar lobe IX	+	FC
											PCC - putamen R	+	FC
											PCC - dlPFC R	–	FC
											PCC - sFG L	+	FC
										AI R	AI R - insula R	–	FC
											AI R - frontoinsula L	+	FC
										dlPFC R	dlPFC R - sPFC R	+	FC
											dlPFC R - dlPFC L	+	FC
											dlPFC R - iPL L	+	FC
											dlPFC R - sFG R	+	FC
Luo et al., 2016^1^	Healthy elderly	32	69.8 (65–75)	MD	passive	24 one-hour group sessions, twice a week	rs-fMRI		ICA		FPN L	+	laterality
											cerebellar network	–	laterality
De Marco et al., 2016	Healthy elderly	46	65.9 (el. criteria >50 y)	MD	active	20 60- to 90-min computer sessions, 5 days a week	rs-fMRI		ICA / seed	PCC R + L	PCC L - precuneus R	+	FC
											PCC L - precuneus R	+	FC
											PCC L - cuneus R	+	FC
											PCC L - cuneus L	+	FC
											PCC L - cuneus L	+	FC
											PCC L - iPL R	+	FC
											PCC L - mFG R	+	FC
											PCC L - PCC R	+	FC
											PCC L - iTG R	+	FC
											PCC L - angular gyrus L	+	FC
											PCC L - parahippocampal gyrus L	–	FC
											PCC R - parahippocampal gyrus L	+	FC
											PCC R - sPL L	+	FC
											PCC R - cuneus L	+	FC
											PCC R - precuneus L	+	FC
											PCC R - sTG L	+	FC
Li et al., 2016^1^	Healthy elderly	54	70 (el. criteria 65–75 y)	MD / SD (reasoning)	active	24 one-hour group sessions, twice a week	rs-fMRI		whole-brain		paracentral lobe	–	functional entropy
											iFG	+	time-domain entropy
											medial sFG^b^	+	time-domain entropy
											thalamus^b^	+	time-domain entropy
											cuneus^b^	+	time-domain entropy
De Marco et al., 2018	MCI	37	73.5 (no range given)	MD	active	20 60- to 90-min computer sessions, 5 days a week	rs-fMRI		ICA	DMN, FPN, visual network	sPL	+	FC
											sPL	+	FC
											iPL	+	FC
Suo et al., 2016^b^	MCI	51	70.1 (el. criteria ≥55 y)	MD	active	52 45-min group sessions, twice a week	rs-fMRI		seed	PCC	PCC - sFG L	–	FC
											PCC - ACC	–	FC
										HC	HC - sFG L	+	FC
Barban et al., 2017^b^	AD / MCI / healthy elderly	26 / 26 / 29	75.5 / 72.2 / 70.5 (el. criteria >60 y)	MD	active	24 one-hour computer sessions, twice a week	rs-fMRI		ICA / whole-brain		precuneus - DMN	+	FC
											medial sFG - DMN *(MCI* vs. *healthy elderly)*	–	FC
											posterior medial temporal lobe L - DMN *(AD)*	–	FC
									graph		orbito-frontal region R *(MCI)*	+	betweenness centrality
											vermis *(MCI)*	–	betweenness centrality
											anterior cingulum R *(AD)*	+	betweenness centrality
									NBS		thalamus L - hippocampus L *(MCI)*	–	FC
											thalamus R - globus pallidus R *(MCI)*	–	FC
											cerebellum R - cuneus R *(MCI)*	–	FC
											calcarine cortex L + R - medial temporal lobe L	+	FC
Diez-Cirarda et al., 2016	PD	30	66.9 (el. criteria 45–75 y)	MD	active	36 one-hour computer sessions, three days a week	rs-fMRI		ROI-to-ROI		iTG L - dlPFC R	+	FC
											iTG L - dlPFC L	+	FC
							tr-fMRI	memory paradigm	whole-brain		mTG L	+	FAct
De Giglio et al., 2016^b^	MS	24	41.9 (el. criteria 18–50 y)	MD	passive	40 30-min computer sessions, five days a week	rs-fMRI		seed	thalamus	thalamus - vermis	–	FC
											thalamus - dlPFC L	–	FC
											thalamus - cerebellum L + R	–	FC
											thalamus - PCC L + R	+	FC
											thalamus - precuneus L + R	+	FC
											thalamus - lPC L + R	+	FC
Bonavita et al., 2015^a,b^	MS	32	47.7 (31–60)	MD	active	16 50-min computer sessions, twice a week	rs-fMRI		ICA		precuneus/iPL - DMN	+	FC
											PCC - DMN	+	FC
Parisi, Rocca, Mattioli, et al., 2014^2^	MS	20	45.8 (25–64)	MD	passive	36 one-hour computer sessions, three times a week	rs-fMRI		seed	ACC (MNI 4, 40, 14)	ACC - iTG	+	FC
											ACC - iPL	+	FC
Filippi et al., 2012^2^	MS	20	45.8 (25–64)	MD	passive	36 one-hour computer sessions, three times a week	rs-fMRI		whole-brain		PCC/precuneus	+	synchronization
											iPL	+	synchronization
											ACC	+	synchronization
											dlPFC	+	synchronization
							tr-fMRI	Stroop test	whole-brain		PCC/precuneus^b^	+	FAct
											dlPFC^b^	+	FAct
Cerasa et al., 2013	MS	23	32.7 (no range given)	MD	active	12 one-hour on-site computer sessions, twice a week	tr-fMRI	paced visual serial addition test	ROI	ACC L + R, lateral premotor cortex, dlPFC, ventrolateral PFC, sPL, iPL, sTG, mTG, thalamus, caudate, cerebellum	sPL	+	FAct
											cerebellum crus I	+	FAct
Campbell et al., 2016^b^	MS	38	47.4 (32–62)	MD	active	18 45-min computer sessions, three times a week	tr-fMRI	n-back	whole-brain		supramarginal gyrus	+	FAct
											angular gyrus	+	FAct
											frontal L *at 12-week follow-up*	+	FAct
											temporoparietal R *at 12-week follow-up*	+	FAct
											prefrontal L + R *at 12-week follow-up*	+	FAct
											temporoparietal R *at 12-week follow-up*	+	FAct
Kim et al., 2017	Healthy elderly	27	71.4 (64–77)	SD (cognitive control)	passive	24 one-hour computer sessions, three times a week	tr-fMRI	multi-source interference task	whole-brain		precentral gyrus R	+	FAct
											supramarginal gyrus R	+	FAct
											postcentral gyrus R	+	FAct
											precuneus R	+	FAct
											anterior insula L	+	FAct
Kuhn et al., 2017^a^	Healthy elderly	53	69 (62–78)	SD (inhibition)	active and passive	56 15-min daily sessions on tablet	tr-fMRI	stop signal task	ROI	iFG R/anterior insula	iFG R/AI	–	FAct
Ross et al., 2018	Healthy elderly	37	70.5 (el. criteria ≥65 y)	SD (processing speed / attention)	active and passive	10 one-hour computer sessions across five weeks	tr-fMRI	useful field of view	ROI	cluster activations during task at pretest: ACC, AI, dlPFC, iPL, supplemental motor area, thalamus, temporoparietal junction, visual cortex	AI	–	FAct
											supplemental motor area	–	FAct
							rs-fMRI		ROI-to-ROI (see above)		increased mean network connectivity	+	FC
											AI - ACC	+	FC
											AI - visual cortex	+	FC
											AI - supplemental motor area	+	FC
											dlPFC - supplemental motor area	+	FC
Lebedev et al., 2018^b^	Healthy elderly	53	69.2 (65–75)	SD (WM)	active	20 40-min computer sessions over four weeks	tr-fMRI	n-back, complex visuospatial reasoning	graph		global	+	modularity
									whole-brain		FPN - SMN, DMN	–	FC
Lin et al., 2016	MCI	21	73 (el. criteria ≥60 y)	SD (processing speed)	active	24 one-hour computer sessions, four times a week	rs-fMRI		ICA		R HC - iFG	+	FC
											R HC - lateral mFG	+	FC
											R HC - medial sFG	+	FC
											R HC - sPL	+	FC
											L HC - iFG	+	FC
											L HC - mFG	+	FC
Huntley et al., 2017	Mild AD	30	79.8 (65–88)	SD (WM)	active	18 computer sessions of 30 trials over eight weeks	tr-fMRI	digit span	ROI	dlPFC L, dlPFC R, parietal cortex L, parietal cortex R	dlPFC R	–	FAct
											parietal cortex L	–	FAct

Studies using the same sample are indicated with the same number in superscript. *In the absence of report on an age range in the study article, the study specific eligibility criteria (el. criteria) concerning age are provided. ^a^ These studies reported only on main effects instead of interaction effects. ^b^ These studies are not included in the Figures, since no anatomical coordinates were reported

“+” Indicates increases, “- “indicates decreases post-training

*Abbreviations* per column - *AD* Alzheimer’s disease, *MCI* mild cognitive impairment, *MS* multiple sclerosis, *PD* Parkinson’s disease, *CG* control group, *CT* cognitive training, *rs-fMRI* resting-state fMRI, *tr-fMRI* task-related fMRI, *ICA* independent component analysis, *ROI* region-of-interest, *NBS* network-based statistic, *ACC* anterior cingulate cortex, *AI* anterior insula, *dlPFC* dorsolateral prefrontal cortex, *DMN* default mode network, *FPN* frontoparietal network, *HC* hippocampus, *iFG* inferior frontal gyrus, *iPL* inferior parietal lobe, *iTG* inferior temporal gyrus, *lPC* lateral parietal cortex, *mFG* middle frontal gyrus, *mTG* middle temporal gyrus, *PCC* posterior cingulate cortex, *sFG* superior frontal gyrus, *sPFC* superior prefrontal cortex, *sPL* superior parietal lobe, *sTG* superior temporal gyrus, *L* left, *R* right, *FAct* functional activity, *FC* functional connectivity

### Quality Assessment

Table 2 shows the quality assessment results. We classified the Diez-Cirarda et al. (2016) paper after re-evaluation as ‘Good’ as the main RCT aspects were present and clearly reported on, and we classified the Lin et al. (2016) paper after re-evaluation as ‘Fair’ as both the blinding and the randomization procedure were not reported on. A major limitation across most studies was the lack of blinding for participants and/or for assessors. The sample size was generally low without clear power calculations to support the sample size, probably due to the exploratory nature of the studies. In general, the neuroimaging methodology was clearly reported on. Concerning competing interests, in the study by Diez-Cirarda et al. (2016), two co-authors were reported to be copyright holders of the studied intervention. Three studies did not report on competing interests (De Marco et al., 2016; Lebedev et al., 2018; Parisi, Rocca, Valsasina, et al., 2014). The other studies reported to have no competing interests.

**Table 2:** Assessment of individual studies on trial and neuroimaging quality

	A. Trial Quality Assessment														B. Neuroimaging quality assessment					Overall assessment
Study	1	2	3	4	5	6	7	8	9	10	11	12	13	14	15	16	17	18	19	
Cao et al., 2016	Y	Y	Y	N	Y	Y	N	N	N	NA	Y	Y	CD	Y	Y	Y	Y	Y	Y	Fair
Luo et al., 2016	Y	Y	Y	N	Y	Y	N	N	N	NA	Y	Y	CD	Y	Y	Y	Y	Y	Y	Fair
De Marco et al., 2016	N	NR	NR	N	NR	Y	Y	Y	NR	NA	Y	NR	CD	N	Y	Y	NR	Y	Y	Poor
Li et al., 2016	Y	Y	Y	N	Y	Y	N	N	N	NA	Y	Y	CD	Y	Y	Y	NR	Y	Y	Fair
De Marco et al., 2018	N	N	NR	NR	NR	Y	N	N	Y	Y	Y	N	CD	NR	Y	Y	NR	Y	Y	Poor
Suo et al., 2016	Y	Y	NR	Y	Y	Y	Y	Y	Y	Y	Y	Y	Y	Y	Y	Y	NR	Y	Y	Good
Barban et al., 2017	Y	Y	Y	NA	Y	Y	Y	NR	NR	Y	Y	NR	CD	NR	Y	Y	Y	Y	Y	Fair
Diez-Cirarda et al., 2016	Y	Y	NR	N	Y	Y	Y	Y	Y	Y	Y	Y	Y	Y	Y	Y	NR	Y	Y	Good
De Giglio et al., 2016	Y	Y	Y	N	Y	CD	Y	NR	NR	Y	Y	N	CD	Y	Y	Y	Y	Y	Y	Fair
Bonavita et al., 2015	N	NR	NR	NR	NR	CD	Y	NR	NR	Y	Y	NR	CD	NR	Y	NR	NR	Y	Y	Poor
Parisi, Rocca, Valsasina, et al., 2014	Y	Y	NR	Y	Y	Y	Y	Y	NR	Y	Y	N	CD	Y	Y	Y	NR	Y	N	Fair
Filippi et al., 2012	Y	Y	NR	Y	Y	Y	Y	Y	NR	Y	Y	N	CD	Y	Y	Y	NR	Y	Y	Good
Cerasa et al., 2013	Y	Y	Y	Y	Y	Y	Y	Y	NR	Y	Y	N	CD	Y	N	Y	NR	Y	Y	Good
Campbell et al., 2016	Y	Y	Y	N	N	Y	Y	Y	Y	Y	Y	NR	Y	Y	N	Y	NR	Y	Y	Fair
Kim et al., 2017	N	N	NR	NA	NR	Y	Y	Y	NR	NR	Y	NR	CD	Y	Y	Y	NR	Y	Y	Poor
Kuhn et al., 2017	Y	Y	Y	NR	NR	Y	Y	Y	NR	NR	Y	Y	CD	Y	Y	Y	NR	Y	Y	Fair
Ross et al., 2018	Y	NR	Y	Y	Y	Y	Y	N	Y	Y	N	NR	CD	Y	Y	Y	NR	Y	Y	Fair
Lebedev et al., 2018	Y	Y	NR	Y	NR	Y	N	Y	NR	N	Y	NR	CD	Y	Y	Y	Y	Y	N	Fair
Lin et al., 2016	Y	NR	NR	N	Y	Y	Y	Y	Y	Y	Y	Y	CD	Y	Y	Y	NR	Y	Y	Fair
Huntley et al., 2017	Y	Y	NR	N	N	Y	Y	Y	NR	NR	Y	Y	Y	Y	Y	Y	NR	Y	NR	Fair

Quality assessment criteria (see Supplementary Material 2 for elaborate description): 1 = randomized (controlled) trial; 2 = adequate randomization; 3 = concealed treatment allocation; 4 = participant and assignment provider blinding; 5 = assessor blinding, 6 = similar group baseline characteristics; 7 = drop-out ≤20%; 8 = differential drop-out ≤15%; 9 = high protocol adherence; 10 = no other interventions; 11 = valid and reliable assessments; 12 = sufficient sample size; 13 = prespecified outcomes and analyses; 14 = intention-to-treat analysis; 15 = clear neuroimaging protocol; 16 = motion-corrected functional images; 17 = equal group motion parameters; 18 = clear neuroimaging analyses; 19 = multiple comparison correction. Trials were coded ‘Poor’ if they adhered to <10 criteria, ‘Fair’ at 10–13 criteria and ‘Good’ at >13 criteria. *Abbreviations: CD* cannot determine, *N* no, *NA* not available, *NR* not reported, *Y* yes

### Results of Individual Studies

**Fig. 2:**
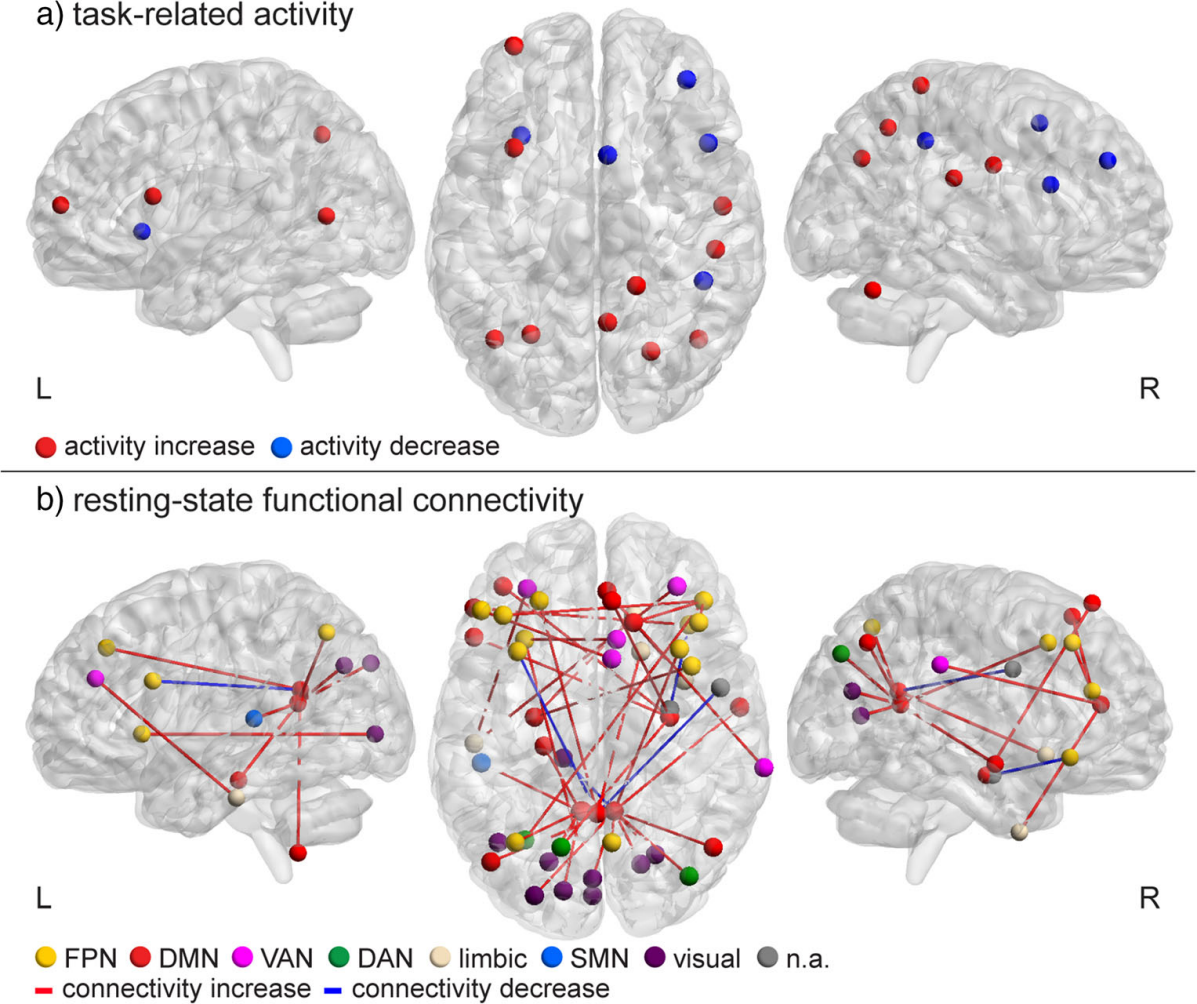
Overview of the findings of all included studies, irrespective of population or training type, that reported coordinates of brain regions with CT-induced alterations. Each dot represents a single alteration in activity (panel **a**) or connectivity (in which both the seed and connected region are displayed; panel **b**). In panel **b**, the seed and connected regions are classified by resting-state network (parcellation according to Yeo et al., 2011; Choi et al., 2012; Buckner et al., 2011) to illustrate within- and between-network connectivity changes. The side views show intra-hemispheric connections. Details about these studies are listed in Table 1. *Abbreviations – FPN: frontoparietal network; DMN: default mode network; VAN: ventral attention network; DAN: dorsal attention network; SMN: somatomotor network; n.a.: no network assigned*

The results of individual studies and methodological details are summarized in Table 1. The results are divided into studies on multi-domain training and single-domain training. A graphical presentation of the findings is shown in Fig. 2.

#### Multi-Domain Training

##### In Healthy Elderly

In healthy elderly, multi-domain training increased functional connectivity of the posterior cingulate cortex (PCC) with other DMN regions (Cao et al., 2016; De Marco et al., 2016) and within-network connectivity of the FPN and salience network (Cao et al., 2016). Cao et al. (2016) additionally reported an increased training-related anti-correlation between the DMN and FPN. In the same dataset, Luo et al. (2016) showed increased laterality of the *left* FPN –an increased confinement of the FPN to the left hemisphere– after CT, which was interpreted as an increase in information processing efficiency. Li et al. (2016) assessed brain entropy in this dataset comparing the multi-domain training with an additional single-domain training and an active control condition (for a detailed description of entropy measures, see T. Li et al., 2016). Both the multi-domain and single-domain training counteracted the age-related increase in whole-brain asynchrony and decrease in spontaneous brain activity. Additionally, the multi-domain training significantly reduced the age-related decrease in lateralization of entropy measures, while single-domain training did not.

##### In Neurodegenerative Diseases

Five studies applied a specific multi-domain training (i.e., ‘RehaCom’) in individuals with MS with variable length (12–36 h). Using independent component analysis on resting-state fMRI scans, RehaCom training increased resting-state functional connectivity within the DMN, mainly in the posterior, parieto-occipital DMN regions (Bonavita et al., 2015), which correlated with a lower post-treatment interference on the Stroop task. Another study showed that RehaCom led to increased or stable resting-state activity fluctuations of salience network, FPN, and DMN areas relative to decreased fluctuations in the control group (Filippi et al., 2012). In this study, task-related activation of the dorsolateral prefrontal cortex (dlPFC) and PCC during the interference condition of the Stroop task was also increased, correlating with performance on a working memory task (paced auditory serial addition test), but no information was provided on the direction of these specific correlations. On the same dataset, Parisi and colleagues (Parisi, Rocca, Valsasina, et al., 2014) reported increased resting-state functional connectivity between the anterior cingulate cortex (ACC) and inferior parietal lobe after RehaCom training that was related to improved performance on a working memory task.

RehaCom training in MS induced increased task-related activation during a working memory task of a temporo-parietal region (Campbell et al., 2016), and in the superior parietal and posterior cerebellar lobe; the latter correlated positively with post-training Stroop interference task performance (lobe VI; Cerasa et al., 2013). After a 12-week follow-up period, task-related activity of the temporal-parietal and additional frontal and prefrontal regions was still higher compared with the control group (Campbell et al., 2016).

A different multi-domain training paradigm in MS resulted in increased resting-state functional connectivity between the thalamus seed and the PCC, precuneus and lateral parietal cortex, and decreased connectivity between the thalamus and the left dlPFC, the vermis, and bilateral cerebellar cortical regions (De Giglio et al., 2016).

In patients with amnestic MCI, multi-domain training decreased functional connectivity of the superior frontal gyrus and ACC with the PCC, a core area of the DMN, but increased connectivity of the hippocampus with the superior frontal gyrus (Suo et al., 2016). Another multi-domain training increased the within-DMN connectivity in MCI patients (De Marco et al., 2018) but did not alter connectivity within the FPN or visual network. The study from Barban et al. (2017) in MCI patients reported decreased functional connectivity of the medial superior frontal gyrus with the DMN after multi-domain training and –using network-based statistics analysis (Zalesky, Fornito, & Bullmore, 2010)– decreased connectivity within a subnetwork consisting of subcortical areas, the cerebellum, and temporal and occipital areas. They additionally reported increased betweenness centrality, i.e., the importance of a particular brain area in long-range network communication (Sporns, 2014), of the orbitofrontal cortex, and decreased betweenness centrality of the cerebellar vermis. Conversely, in the same study multi-domain training in AD patients increased the spatial extent of the DMN, increased the functional connectivity of the network-based statistics subnetwork and increased betweenness centrality of the ACC. The authors therefore concluded that multi-domain training had different effects on the brain in MCI and AD, despite similar cognitive improvements. It must be noted, however, that MCI patients improved mainly on memory tasks while AD patients showed an improvement in attention.

In PD patients, an integrative multi-domain training resulted in increased resting-state functional connectivity between the left inferior temporal lobe and bilateral dlPFC and increased activation of the left middle temporal lobe during a memory task (Diez-Cirarda et al., 2016).

##### Synthesis of Results

Results from multi-domain training studies coherently suggest that it counteracts age-related or disease-related network dysfunction by increasing the within-network connectivity –predominantly reported in the DMN– and the degree of anti-correlation between networks, in particular between the FPN and DMN, which has been related to better cognitive functioning (Kelly et al., 2008; Baggio et al., 2015; Hampson, Driesen, Roth, Gore, & Constable, 2010). Studies on within-DMN connectivity alterations in MCI and AD patients showed mixed results, however, with both increased and decreased connectivity in this resting-state network. CT increased task-related activity across the brain, suggesting an increased neural effort, although this was only studied in MS and PD patients.

#### Single-Domain Training

##### In Healthy Elderly

Processing speed/attention training induced decreased activation in the anterior insula and supplementary motor area during an in-scanner ‘useful field of view’ task (Ross et al., 2018) – a visual processing speed and attention task (Wood & Owsley, 2014). This reduction in brain activation was significantly different compared with no-contact controls, while no training-induced activation differences were found relative to an active control group. In this study, resting-state functional connectivity of areas that showed activation during the task at baseline increased significantly after the experimental training compared with *both* control groups. Kuhn et al. (2017) compared inhibition training to 1) multi-domain training on a mobile application and 2) a passive control condition, and found decreased activation of the right inferior frontal gyrus/anterior insula during an in-scanner stop-signal task after inhibition training only, although this effect did not reach significance in the interaction model (i.e., relative to the control groups). A cognitive control training *increased* activation of right frontoparietal regions and the left anterior insula during an interference control task (Kim et al., 2017). These results were associated with cognitive performance improvement, mainly on the Stroop color-word interference task.

Working memory training increased brain-network segregation during an *n*-back and a visuospatial reasoning task as shown by increased whole-brain modularity and reduced connectivity between the FPN and both the DMN and sensorimotor network after training (Lebedev et al., 2018). Interestingly, before training modularity was positively associated with working memory performance, but not with the complex reasoning task. The authors argued that the working memory training potentially induced an increased modular organization that is beneficial for specific abilities such as working memory, but deleterious for complex cognitive tasks.

##### In Neurodegenerative Diseases

In amnestic MCI patients, a visuospatial speed of processing training resulted in increased resting-state functional connectivity within the DMN (Lin et al., 2016), while increased FPN connectivity did not differ significantly from the active control group. An adaptive WM training in early AD resulted in decreased averaged post-training activity of the right dlPFC and left parietal cortex (Huntley et al., 2017).

##### Synthesis of Results

Single-domain training paradigms increased functional connectivity within networks and reduced connectivity between networks, similar to connectivity alterations after multi-domain training, but only two studies performed functional connectivity analyses. These paradigms additionally induced reductions in task-related activations in both healthy elderly and an MCI population, which is generally interpreted as an increased efficiency of neural resources needed for the task at hand (Thompson, Waskom, & Gabrieli, 2016; Clark et al., 2017). Reduced activation may additionally be related to the fact that the content of the single-domain CT was identical (Huntley et al., 2017; Ross et al., 2018) or highly similar (Kuhn et al., 2017) to the in-scanner task, thus inducing practice effects. Accordingly, one study that used an in-scanner task that was *dissimilar* to the CT found *increased* training-related activity (Kim et al., 2017). The quality rating of this study was, however, poor so these results should be interpreted with caution.

## Discussion

This paper systematically reviews studies that investigated the influence of CT through repeated cognitive engagement (i.e., process-based training) on task-related activity and resting-state functional connectivity using fMRI in both healthy populations and neurodegenerative diseases. Our results show that all investigated CT paradigms led to changes in brain activation during task performance and resting-state functional connectivity. There are three main conclusions to our research questions that can be drawn from this literature overview: 1) CT induced both increases and decreases in task-related activity, mostly in fronto-parietal brain areas, without a clear influence of the targeted cognitive domains; 2) multi-domain CT was consistently reported to counteract dysfunctional connectivity patterns in cognitive brain networks that are generally associated with aging or neurodegenerative diseases; 3) methodological heterogeneity between studies limits our ability to statistically compare findings and study disease- or training-specific neural alterations. Below we consider the implications of our findings and critically discuss methodological issues to guide future research.

### Task-Related Activation Studies: Training-Induced Improvement in Increased Neural Efficiency, or Increased Effort

The majority of single-domain studies and four multi-domain CT studies assessed training-induced alterations in task-related brain activation. We observed that alterations were most frequently reported in frontoparietal areas, probably driven by the fact that the FPN was a network of interest in most studies (see “Limitations and recommendations for future research” section). Three single-domain studies found *decreased* task-related activity after CT (Huntley et al., 2017; Kuhn et al., 2017; Ross et al., 2018), while one single-domain study and all multi-domain studies found *increased* task-related activity (Kim et al., 2017; Diez-Cirarda et al., 2016; Filippi et al., 2012; Cerasa et al., 2013; Campbell et al., 2016). These results of both increased and decreased activity are in line with a meta-analysis in healthy elderly that similarly reported on functional activity alterations predominantly in areas of the FPN (Duda & Sweet, 2019). A review on working memory training in healthy populations reported predominantly post-training regional activity decreases, while increased activity was more scarcely reported (Buschkuehl, Jaeggi, & Jonides, 2012). An interesting distinction was found in one study that the review described; reporting decreased post-CT activity in young adults but increased activity in elderly suggesting different processes of plasticity (Dahlin, Neely, Larsson, Backman, & Nyberg, 2008). Another similar review in healthy populations also reported both increased and decreased brain activity after CT, possibly related to task selectivity, improved efficiency or more automatic processing due to CT (Taya, Sun, Babiloni, Thakor, & Bezerianos, 2015). We hypothesize that either training-induced neural efficiency *or* neural effort can account for the bi-directionality of the results, although in-scanner task characteristics may also be relevant.

**Fig. 3:**
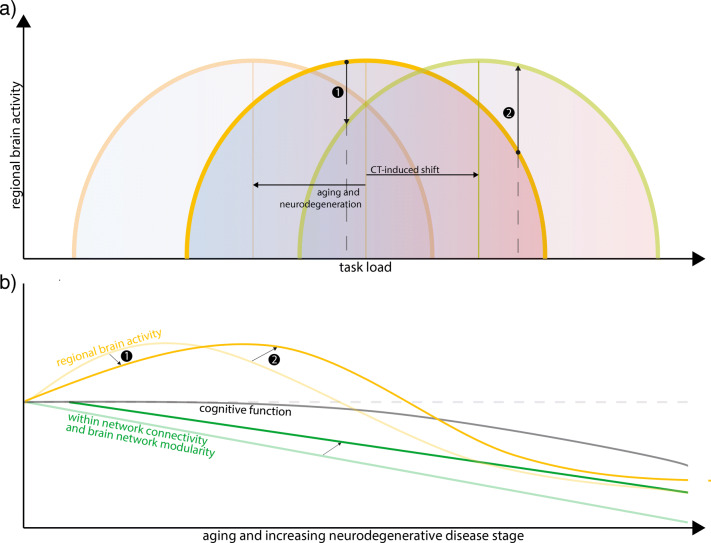
Working model. **a** The inverted *U*-shaped association between regional brain activity during task performance and task load. Aging and neurodegenerative diseases lead to a shift of the curve to the left, while CT seems to induce the opposite, illustrated by the horizontal arrows. Consequently, at the same task load different neural resources are needed/used. **b** The association between task-related brain activity and network connectivity and modularity at increasing age or disease stage. The arrows indicate the suggested effect of CT at different stages of aging or disease. Both in panel (**a**) and (**b**), **(1)** indicates training-induced hypo-activity associated with neural efficiency: either a) tasks with lower demand can be performed more efficiently through cognitive training (panel **a**), or b) CT (partially) restores compensatory hyper-activity that is associated with early stages of aging and neurodegenerative diseases to a more ‘healthy’ state (panel **b**); **(2)** indicates that CT leads to hyper-activity that is associated with increased effort and which is needed to successfully fulfill a task with a high cognitive demand

Decreased task-related activity after training suggests an increase in efficiency, i.e., reduced use of neural resources. This result was described only in healthy elderly samples and fits well with the literature on compensatory mechanisms of increased neural resource use to uphold ‘normal’ cognitive task performance in healthy aging (Reuter-Lorenz & Cappell, 2008; Reuter-Lorenz & Park, 2014; Cabeza, 2002; Davis, Dennis, Daselaar, Fleck, & Cabeza, 2008). It is generally accepted that aging-related decline in brain ‘fitness’ due to, for example, decreased within-network connectivity, increased neural noise, or dedifferentiation of task-positive and task-negative neural networks is compensated for by mechanisms such as regional over-activation, a posterior-to-anterior shift in brain activity, and decreased asymmetry of hemispheric activity (Reuter-Lorenz & Park, 2010; Festini, Zahodne, & Reuter-Lorenz, 2018; Cabeza et al., 2018). Our results may therefore indicate that the increased efficiency after single-domain training reverses age-related compensatory mechanisms of increased neural effort while maintaining cognitive performance (illustrated by (1) in Fig. 3, panel a and b).

Four studies in patients with PD or MS reported an *increase* in post-training activity during task performance. This seems to reflect an increase in neural effort, which is in contrast with the increase in efficiency as described above – but may be related to differences between healthy and non-healthy populations. Theories on compensatory mechanisms with healthy aging assume that significant neural remodeling occurs, while at the behavioral level performance remains relatively unimpaired (Festini et al., 2018). This mechanism has also been described in early stages of neurodegenerative diseases in which task performance is still comparable to age-matched healthy controls (Trujillo et al., 2014; Gerrits et al., 2015; Audoin et al., 2003; Lopez-Gongora et al., 2015). Indeed, in a recent study, working memory CT showed similar task-related activity decrease in healthy adults and a population of early-stage cognitively healthy MS patients (Aguirre et al., 2019). Cognitive performance in MS and PD patients is, however, generally impaired (Bosboom, Stoffers, & Wolters, 2004; Chiaravalloti & DeLuca, 2008) and compensatory mechanisms as described above may at later disease stage no longer be sufficient for these patients. Indeed, task-related neural activity is hypothesized to follow an inverted *U*-shaped curve both in healthy adults and elderly subjects (Cabeza et al., 2018), in patients suffering from MS (Schoonheim, Geurts, & Barkhof, 2010) and neuropsychiatric disorders (van Velzen, Vriend, de Wit, & van den Heuvel, 2014), so that when task demands become too high, compensatory hyperactivity fails resulting in relative hypo-activity and impaired performance. In the reviewed studies on MS and PD patients, we hypothesize that –as baseline cognitive performance was impaired in these studies– training resulted in a shift of the *U*-shaped curve to the right thus counteracting the relative hypo-activity at baseline, and improved performance (illustrated as (2) in Fig. 3). There is, however, no information on the baseline cognitive performance of the MS and PD samples relative to healthy controls.

Differences in task demand and familiarity may also have impacted the observed outcomes. In accordance with the literature on compensatory brain processes (Reuter-Lorenz & Cappell, 2008; Cabeza et al., 2018), variation in task load difficulty between task-related fMRI studies may have induced different results with less demanding fMRI tasks such as a digit span task (Huntley et al., 2017) inducing increased efficiency of neural resources, while complex tasks such as the multi-source interference task (Kim et al., 2017) induced increased brain activity. Moreover, some studies using fMRI task paradigms that were also part of the CT paradigm, e.g., in Huntley et al. (2017) and Ross et al. (2018), reported post-training activity *decreases,* while studies using an fMRI task that was less similar to the tasks being trained (such as in the multi-domain training studies) showed *increased* post-training activity. In line with theories of neural efficiency (Reuter-Lorenz & Cappell, 2008) and neural scaffolding (Reuter-Lorenz & Park, 2014), this may indicate increased neural efficiency by familiarity of the task paradigm –i.e., having repeatedly performed one specific task– or increased scaffolding by being able to address compensatory neural resources, respectively.

### Counteracting Age- or Disease-Related Neural Network Dysfunctions

The brain is organized into several segregated functional and structural networks that facilitate the execution of complex functions (Wig, 2017; Fox et al., 2005; Seeley et al., 2007). Aging and neurodegenerative diseases lead to reductions in the connectivity *within* networks and decreased segregation of (i.e., increased connectivity between) these networks (Spreng, Sepulcre, Turner, Stevens, & Schacter, 2013; Grady, Sarraf, Saverino, & Campbell, 2016; Joo, Lim, & Lee, 2016; Hohenfeld, Werner, & Reetz, 2018; Damoiseaux, 2017). Our review shows that relative to a control condition, CT consistently induced neural alterations that counteracted these age- and disease-related connectivity patterns. This was particularly evident for studies on multi-domain training. First, CT increased intra-network functional connectivity. This effect was most frequently reported within the DMN, but also for the FPN, DAN/VAN connectivity and in functional connectivity of the hippocampus and thalamus. Additionally, CT enhanced the degree of network *segregation,* evident from increases in the anti-correlation between task-negative (i.e., DMN) and task-positive networks (FPN or DAN), or a training-induced increase in whole-brain modularity. Enhanced segregation is associated with better cognitive functioning in the network literature (Damoiseaux, 2017).

Two reports specifically addressed age-related neural alterations and described a long-term effect of CT on resting-state fMRI-derived indices of neural network laterality (Luo et al., 2016) and brain entropy (Li et al., 2016), even a year after training. Similarly, a recent study in patients with MCI found increased spontaneous regional brain activity during resting-state (Li et al., 2019). Taken together, the results thus seem to indicate a restorative effect of CT on aging and neurodegeneration-induced changes in neural network organization (see Fig. 3, panel b). This seems mainly applicable to multi-domain training. It should be noted that only a single study enrolled both a healthy and a non-healthy population; it therefore remains speculative whether CT indeed restores brain network connectivity of patients with neurodegenerative diseases to healthy control levels.

### Limitations and Recommendations for Future Research

The main shortcoming of the reviewed literature is the heterogeneity in type of training, imaging methodology, in-scanner task paradigm and analysis method, which did not allow us to do statistical comparisons through meta-analyses. The reviewed studies all show that CT induces changes in brain activity and connectivity that are not localized to specific brain regions. Our systematic review therefore does not seem to confirm an earlier reported assumption that neural alterations are specific to a particular type of CT (Buschkuehl et al., 2012) – although there are some general differences between multi- and single-domain training. It remains, however, unclear if any population- or training-specific effects exist. There is a significant body of literature on functional activity and connectivity changes after strategy-based CT that show similar findings as the results from this review. For instance, mnemonic strategy CTs increased activity of fronto-parietal and temporal regions in individuals suffering from MCI and increased functional connectivity within cognitive networks in healthy elderly (Chapman et al., 2015; Chapman, Spence, Aslan, & Keebler, 2017; Hampstead, Stringer, Stilla, Giddens, & Sathian, 2012; Hampstead, Stringer, Stilla, & Sathian, 2019; Simon et al., 2019). These studies were, however, beyond the scope of this review. An additional issue impacting the results of this review is that there is a likelihood of publication bias given the lack of negative results in this review sample. Future research should focus on statistically comparing training packages and types, population-specific CT effects and quantify publication bias. Likewise, our quality assessment identified several low-powered, non-blinded, or non-randomized studies, which substantiates the need for future studies with unified CT paradigms and analysis methods to enhance comparability. The use of healthy control samples could additionally highlight differential effects of CT on healthy and non-healthy populations and shed light on possible restorative effects that CT might induce. One recently published study in a small group of individuals with MS showed for example that hyper-activation relative to healthy controls at baseline was partially normalized after CT (Bonzano et al., 2018).

An important methodological limitation of the reviewed studies is the fact that the majority of studies focused on alterations of the activity or connectivity of a single or only a few brain regions using ROI- or seed-based approaches. As a consequence, the selection of the ROI or seed determines the observed patterns and (slight) shifts in location may already lead to different results (Li, Guo, Nie, Li, & Liu, 2009). Moreover, ROI- or seed-based approaches are based on the assumption that cognitive functions are related to discrete brain regions and thus focus on just a piece of the puzzle (Turk-Browne, 2013). By exclusively focusing on particular ROIs or seed regions, other potentially interesting and meaningful brain activity and connectivity patterns might have been missed. Neural correlates of CT were, for example, reported mainly in cognitive brain networks, but the majority of the ROIs or seed regions were located within these networks which biases the reported effects. As higher-order cognitive functions typically require the integration of multiple brain processes by having large-scale networks interact dynamically, instead of relying on independent, localized processes (Menon, 2011; Bullmore & Sporns, 2009; Bassett & Sporns, 2017), whole-brain, integrative approaches are indispensable in the assessment of neural CT correlates.

Improvements in computational power in neuroimaging and its analyses have led to a network approach that can grasp the complexity of behavior including cognitive functions (Ferguson, Anderson, & Spreng, 2017; Bassett & Sporns, 2017; Stam, 2014). In neuroscience, neural network topology and dynamics are strongly correlated with cognitive functions (Douw et al., 2011; Langer et al., 2012; Camicioli et al., 2009). An example of an integrative approach is offered by a single study in this review that used modularity as a graph theoretical measure to assess neural network segregation after CT (Lebedev et al., 2018). The results of this study confirm the findings from seed-based connectivity studies in this review of CT-induced enhanced task-positive versus task-negative network segregation during task performance. It has further been advocated that to really understand the neural correlates of executive functioning –and by extension the working mechanism of CT– one needs to study the dynamic and flexible engagement of brain networks (Braun et al., 2015). Interactions between brain regions constantly change during task execution, a process called dynamic network reconfiguration, which is dependent on task demands (Braun et al., 2015; Kitzbichler, Henson, Smith, Nathan, & Bullmore, 2011; Bentivoglio, Baldonero, Ricciardi, De Nigris, & Daniele, 2013). Converging evidence of both integrative and targeted approaches may in the end lead to a better understanding of how CT alters brain function.

Lastly, although most studies show that CT is able to induce either clinical cognitive improvement or lead to changes in brain activity and/or connectivity, few studies report on an actual association between CT-induced alterations in brain function and the change in neuropsychological measures. Yet, from a clinical perspective, it is essential to demonstrate these associations between neural alterations and improvement on neuropsychological measures and, even more so, in measures of everyday function. Furthermore, the organization of brain networks may serve as a predictive biomarker for treatment response to facilitate personalized CT programs (i.e., precision medicine). Multiple studies in neurological and psychiatric populations have shown that the individual variation in pre-training brain morphological and/or network characteristics, e.g., brain modularity or cortical volume, is related to the variability in CT-induced cognitive improvement (Arnemann et al., 2015; Engvig et al., 2012; Gallen et al., 2016; Strangman et al., 2010; Verghese, Garner, Mattingley, & Dux, 2016; Vermeij et al., 2016). In addition, neural activation and functional connectivity after CT reportedly predict the persistence of neuropsychological and behavioral improvement at follow-up testing (Subramaniam et al., 2012, 2014; Parisi, Rocca, Mattioli, et al., 2014).

### Conclusion

There is convincing evidence that cognitive process-based training alters brain activation and connectivity patterns. CT-induced changes occur mainly in neural networks important for cognitive function and seem to counteract dysfunctional activation and connectivity patterns associated with aging and neurodegenerative diseases, either indicative of a restorative or compensatory process – or a combination thereof. In order to improve our understanding of CT-induced neural alterations and the associated cognitive improvement, we advocate a network view of the brain to better comprehend the complex, dynamic changes in the brain induced by training. It is essential to harmonize the methodology and improve trial quality to increase comparability between studies and ultimately enable quantitative meta-analyses. Knowledge of how CT alters the brain network and how this relates to cognitive improvement may ultimately improve CT efficacy and accelerate individualized cognitive training programs.

## Electronic supplementary material

ESM 1 (PDF 363 kb)

## Abbreviations

ACC: Anterior cingulate cortex
AD: Alzheimer’s disease
CT: Cognitive training
DAN: Dorsal attention network
dlPFC: Dorsolateral prefrontal cortex
DMN: Default mode network
(f) MRI: (functional) MRI
FPN: Frontoparietal network
MCI: Mild cognitive impairment
MNI: Montreal Neurological Institute
MRI: Magnetic resonance imaging
MS: Multiple sclerosis
PCC: Posterior cingulate cortex
PD: Parkinson’s disease
ROI: Region-of-interest
VAN: Ventral attention network
