# Supplementary material for: The Effects of Cognitive Training on Brain Network Activity and Connectivity in Aging and Neurodegenerative Diseases: a Systematic Review
Source: Neuropsychol Rev. 2020 Jun 12;30(2):267–86. doi: 10.1007/s11065-020-09440-w (PMC7305076; doi:10.1007/s11065-020-09440-w)
Supplement: Supplementary file 1 — (PDF 363 kb) [file 11065_2020_9440_MOESM1_ESM.pdf]

**Electronic Supplementary Material |** Van Balkom et al - The effects of cognitive training on brain network activity and connectivity in aging and neurodegenerative diseases: a systematic review. In: Neuropsychology Review

**Electronic Supplementary Material 1 – Overview of structured literature search**

Three components were added in the literature search, i.e. a combination of “cognitive training” and related terms, “neuroimaging” and related terms, and exclusion terms based on our eligibility criteria. The search strategy below was used in PubMed and translated to PsycINFO and Embase.

1. *Cognitive training component*

“cognitive training”[tiab] OR “attention training”[tiab] OR “working memory training”[tiab] OR “memory training”[tiab] OR “strategy training”[tiab] OR “processing speed training”[tiab] OR “speed-of-processing training”[tiab] OR “multitasking training”[tiab] OR “multi-tasking training”[tiab] OR “cognitive rehabilitation”[tiab] OR “attention rehabilitation”[tiab] OR “working memory rehabilitation”[tiab] OR “memory rehabilitation”[tiab] OR “strategy rehabilitation”[tiab] OR “processing speed rehabilitation”[tiab] OR “speed-of-processing rehabilitation”[tiab] OR “multitasking rehabilitation”[tiab] OR “multi-tasking rehabilitation”[tiab] OR “cognitive intervention”[tiab] OR “attention intervention”[tiab] OR “working memory intervention”[tiab] OR “memory intervention”[tiab] OR “strategy intervention”[tiab] OR “processing speed intervention”[tiab] OR “speed-of-processing intervention”[tiab] OR “multitasking intervention”[tiab] OR “multi-tasking intervention”[tiab] OR “cognitive enhancement therapy”[tiab] OR “attention enhancement therapy”[tiab] OR “working memory enhancement therapy”[tiab] OR “memory enhancement therapy”[tiab] OR “strategy enhancement therapy”[tiab] OR “processing speed enhancement therapy”[tiab] OR “speed-of-processing enhancement therapy”[tiab] OR “multitasking enhancement therapy”[tiab] OR “multi-tasking enhancement therapy”[tiab] OR “cognitive stimulation”[tiab]

2. *Neuroimaging component*

network*[tiab] OR brain[tiab] OR neural*[tiab] OR “magnetic resonance imaging”[tiab] OR fMRI[tiab] OR MRI[tiab] OR connectivity[tiab] OR neurofunctional[tiab] OR structural[tiab] OR functional[tiab] OR neuroimaging[MeSH]

3. *Exclusion criteria components*

a. Not: psychotherapy[tiab] OR “cognitive behavioral therapy”[tiab] OR “acceptance and commitment therapy”[tiab] OR mindfulness[tiab] OR meditat*[tiab] OR “transcranial magnetic stimulation”[tiab] OR TMS[tiab] OR “transcranial direct current stimulation”[tiab] OR tDCS[tiab] OR “deep brain stimulation”[tiab] OR DBS[tiab] OR “electroconvulsive therapy”[tiab] OR ECT[tiab] OR magnetoencephalo*[tiab] OR electroencephalo*[tiab]
b. No animal studies:
   Not: animals[Mesh] NOT humans[Mesh]
c. Adult studies:
   Not: ("Adolescent"[Mesh] OR "Child"[Mesh] OR "Infant"[Mesh] OR adolescen*[tiab] OR child*[tiab] OR schoolchild*[tiab] OR infant*[tiab] OR girl*[tiab] OR boy*[tiab] OR teen[tiab] OR teens[tiab] OR teenager*[tiab] OR youth*[tiab] OR pediatr*[tiab] OR paediatr*[tiab] OR puber*[tiab]) NOT ("Adult"[Mesh] OR adult*[tiab] OR man[tiab] OR men[tiab] OR woman[tiab] OR women[tiab])

**Electronic Supplementary Material 2 – Quality assessment form of reviewed articles**

| CRITERIA | YES | NO | OTHER (CD, NR, NA)/COMMENT |
|---|---|---|---|
| ***TRIAL QUALITY ASSESSMENT*** | | | |
| 1. Was the study described as randomized, a randomized trial, a randomized clinical trial, or an RCT? | | | |
| 2. Was the method of randomization adequate (i.e., use of randomly generated assignment)? | | | |
| 3. Was the treatment allocation concealed (so that assignments could not be predicted)? | | | |
| 4. Were study participants and providers blinded to treatment group assignment? | | | |
| 5. Were the people assessing the outcomes blinded to the participants' group assignments? | | | |
| 6. Were the groups similar at baseline on important characteristics that could affect outcomes (e.g., demographics, risk factors, co-morbid conditions)? | | | |
| 7. Was the overall drop-out rate from the study at endpoint 20% or lower of the number allocated to treatment? | | | |
| 8. Was the differential drop-out rate (between treatment groups) at endpoint 15 percentage points or lower? | | | |
| 9. Was there high adherence to the intervention protocols for each treatment group? | | | |
| 10. Were other interventions avoided or similar in the groups (e.g., similar background treatments)? | | | |
| 11. Were outcomes assessed using valid and reliable measures, implemented consistently across all study participants? | | | |
| 12. Did the authors report that the sample size was sufficiently large to be able to detect a difference in the main outcome between groups with at least 80% power? | | | |
| 13. Were outcomes reported or subgroups analyzed pre-specified (i.e., identified before analyses were conducted)? | | | |
| 14. Were all randomized participants analyzed in the group to which they were originally assigned, i.e., did they use an intention-to-treat analysis? | | | |
| ***NEUROIMAGING QUALITY ASSESSMENT*** | | | |
| 15. Was the neuroimaging protocol clearly described? | | | |
| 16. Were functional images corrected for motion? | | | |
| 17. Were motion parameters equal between groups? | | | |
| 18. Were the neuroimaging analyses clearly described? | | | |
| 19. Did the authors correct for multiple comparisons by using corrected p-values? | | | |
| **QUALITY RATING (GOOD, FAIR, OR POOR)** | | | |
| **RATER #1:** | | | |
| **RATER #2:** | | | |
| **ADDITIONAL COMMENTS (IF POOR, PLEASE STATE WHY):** | | | |

*CD, cannot determine; NA, not applicable; NR, not reported

**Electronic Supplementary Material 3 – Overview of excluded studies after full-text review**

**Reason of exclusion**

1) **No neurodegenerative disease/healthy elderly population (N=37)**

Bolte et al. (2015); Beauchamp et al. (2016); Bless et al. (2014); Brooks et al. (2016); Caeyenberghs et al. (2016); Clark et al. (2017); Eack et al. (2010); Eack et al. (2016); Fan et al. (2017); Gaab et al. (2006); Hooker et al. (2012); Hooker et al. (2013); Hu et al. (2017); Kuhn et al. (2013); Kable et al. (2017); Keshavan et al. (2017); Lee et al. (2012); Lin et al. (2014); Lorenz et al. (2015); Metzler-Baddeley et al. (2016a, 2016b); Metzler-Baddeley et al. (2017); Miro-Padilla et al. (2019); Ramsay et al. (2018); Schneiders et al. (2011); Schneiders et al. (2012); Schweizer et al. (2013); Subramaniam et al. (2012); Subramaniam et al. (2014); Sun et al. (2016); Takeuchi et al. (2011a); Takeuchi et al. (2011b); Takeuchi et al. (2013, 2014); Takeuchi et al. (2016); Takeuchi et al. (2017); Thompson et al. (2016)

2) **No pre- and post-CT fMRI assessment (N=12)**

Beatty et al. (2015); Colom et al. (2016a); Colom et al. (2016b); Ghio et al. (2018); Jiang et al. (2016); Kim et al. (2015); Mozolic et al. (2010); Nozawa et al. (2015); Raz et al. (2013); Roman et al. (2016); Roman et al. (2017); Vartanian et al. (2013)

3) **No process-based cognitive training intervention (N=18)**

Ceccarelli et al. (2009); Chapman et al. (2015); Chapman et al. (2017); Engvig et al. (2010, 2012); Engvig et al. (2014); Ernst et al. (2018); Hampstead et al. (2012a); Hampstead et al. (2012b); Han et al. (2018a); Han et al. (2017); Han et al. (2018b); Miotto et al. (2014); Miotto et al. (2013); Miotto et al. (2006); Motes et al. (2018); Murphy et al. (2017); Osaka et al. (2012)

4) **Combination intervention (N=4)**

Hotting et al. (2013); Train the Brain Consortium (2017); Yin et al. (2014); Zheng et al. (2015)

5) **Sample size < 20 (N=3)**

Cerasa et al. (2014); Jolles et al. (2013); Kirchhoff et al. (2012)

6) **No control group (N=4)**

Chang et al. (2017); Heinzel et al. (2017); Venza et al. (2016); Wei et al. (2014)

**Electronic Supplementary Material 4 –** Description of the study interventions and re-coding of CT type to single-domain or multi-domain training.

| Study | | Intervention description | Re-coding |
|---|---|---|---|
| Cao et al. 2016 | CT | Multi-domain cognitive training targeting memory, reasoning and problem-solving. | Multi-domain |
| | Control | Wait-list. | Passive |
| Luo et al. 2016 | CT | Multi-domain cognitive training targeting memory, reasoning and problem-solving. | Multi-domain |
| | Control | Wait-list. | Passive |
| De Marco et al. 2016 | CT | Cognitive training with semantic retrieval, logical reasoning, proper names retrieval and speed of processing tasks, and a single-trial final exercise. | Multi-domain |
| | Control | A daily regime of intense social interactions. | Active |
| Li et al. 2016 | CT | Multi-domain cognitive training targeting memory, reasoning and problem-solving *or* cognitive training focusing solely on reasoning. | Multi-domain and single-domain |
| | Control | Lectures about healthy living every two months. | Active |
| De Marco et al. 2018 | CT | Cognitive training with semantic retrieval, logical reasoning, proper names retrieval and speed of processing tasks, and a single-trial final exercise. | Multi-domain |
| | Control | A daily regime of intense social interactions. | Active |
| Suo et al. 2016 | CT | Multi-domain training with fourteen exercises focusing on memory, working memory, speed of processing, executive function and attention. | Multi-domain |
| | Control | Watching documentary clips and answering simple questions about these. | Active |
| Barban et al. 2017 | CT | Cognitive training including memory, executive function, attention, reasoning and ‘other’ domain exercises. | Multi-domain |
| | Control | Name and numeric value entry. | Active |
| Diez-Cirarda et al. 2016 | CT | Cognitive training including attention, memory, language, executive function and social cognition components. | Multi-domain |
| | Control | Occupational therapy (drawing, reading news, constructing) | Active |
| De Giglio et al. 2016 | CT | Cognitive training including memory, attention, visuospatial processing and calculation games. | Multi-domain |
| | Control | No intervention. | Passive |
| Bonavita et al. 2015 | CT | Cognitive training including attention and concentration, plan a day, divided attention, reaction behavior and logical thinking sessions. | Multi-domain |
| | Control | Newspaper reading and explaining the content | Active |
| Parisi et al. 2014 | CT | Cognitive training including attention, information processing and executive function components. | Multi-domain |
| | Control | No intervention. | Passive |
| Filippi et al. 2012 | CT | Cognitive training including attention, information processing and executive function components. | Multi-domain |
| | Control | No intervention. | Passive |
| Cerasa et al. 2013 | CT | Cognitive training including several attention ability and information processing tasks. | Multi-domain |
| | Control | Visuomotor coordination reaction time task. | Active |
| Campbell et al. 2016 | CT | Cognitive training divided in three modules focusing on working memory, visuospatial memory and divided attention. | Multi-domain |
| | Control | No intervention. | Passive |
| Kim et al. 2017 | CT | Cognitive training using five tasks of cognitive control. | Single-domain |
| | Control | No intervention. | Passive |
| Kuhn et al. 2017 | CT | Cognitive training using an inhibition game. | Single-domain |
| | Control | Both a mobile cognitive training application *or* no intervention. | Active and passive |

| | | | |
|---|---|---|---|
| Ross et al. 2018 | CT | Useful field of view training focusing on diverse attention functions. | Single-domain |
| | Control | Both a complex paper-and-pencil training targeting higher level reasoning, recall and executive function, *or* a no intervention condition. | Active and passive |
| Lebedev et al. 2018 | CT | Cognitive training using four tasks of working memory. | Single-domain |
| | Control | Perceptual speed training. | Active |
| Lin et al. 2016 | CT | Cognitive training using five tasks focusing on vision-based speed of processing. | Single-domain |
| | Control | Online crosswords, Sudoku and solitaire games. | Active |
| Huntley et al. 2017 | CT | Cognitive training of adaptive digit span recall using chunking. | Passive |
| | Control | Non-adaptive digit sequence of three digits. | Active |

**References**

Beatty, E. L., Jobidon, M. E., Bouak, F., Nakashima, A., Smith, I., Lam, Q., et al. (2015). Transfer of training from one working memory task to another: Behavioural and neural evidence. *Front Syst Neurosci, 9*, 86, doi:10.3389/fnsys.2015.00086.

Beauchamp, K. G., Kahn, L. E., & Berkman, E. T. (2016). Does inhibitory control training transfer?: Behavioral and neural effects on an untrained emotion regulation task. *Soc Cogn Affect Neurosci, 11*(9), 1374-1382, doi:10.1093/scan/nsw061.

Bless, J. J., Westerhausen, R., Kompus, K., Gudmundsen, M., & Hugdahl, K. (2014). Self-supervised, mobile-application based cognitive training of auditory attention: A behavioral and fmri evaluation. *Internet Interventions, 1*(3), 102-110.

Bolte, S., Ciaramidaro, A., Schlitt, S., Hainz, D., Kliemann, D., Beyer, A., et al. (2015). Training-induced plasticity of the social brain in autism spectrum disorder. *Br J Psychiatry, 207*(2), 149-157, doi:10.1192/bjp.bp.113.143784.

Brooks, S. J., Burch, K. H., Maiorana, S. A., Cocolas, E., Schioth, H. B., Nilsson, E. K., et al. (2016). Psychological intervention with working memory training increases basal ganglia volume: A vbm study of inpatient treatment for methamphetamine use. *Neuroimage Clin, 12*, 478-491, doi:10.1016/j.nicl.2016.08.019.

Caeyenberghs, K., Metzler-Baddeley, C., Foley, S., & Jones, D. K. (2016). Dynamics of the human structural connectome underlying working memory training. *J Neurosci, 36*(14), 4056-4066, doi:10.1523/JNEUROSCI.1973-15.2016.

Ceccarelli, A., Rocca, M. A., Pagani, E., Falini, A., Comi, G., & Filippi, M. (2009). Cognitive learning is associated with gray matter changes in healthy human individuals: A tensor-based morphometry study. *Neuroimage, 48*(3), 585-589, doi:10.1016/j.neuroimage.2009.07.009.

Cerasa, A., Gioia, M. C., Salsone, M., Donzuso, G., Chiriaco, C., Realmuto, S., et al. (2014). Neurofunctional correlates of attention rehabilitation in parkinson's disease: An explorative study. *Neurol Sci, 35*(8), 1173-1180, doi:10.1007/s10072-014-1666-z.

Chang, L., Lohaugen, G. C., Andres, T., Jiang, C. S., Douet, V., Tanizaki, N., et al. (2017). Adaptive working memory training improved brain function in human immunodeficiency virus-seropositive patients. *Ann Neurol, 81*(1), 17-34, doi:10.1002/ana.24805.

Chapman, S. B., Aslan, S., Spence, J. S., Hart, J. J., Jr., Bartz, E. K., Didehbani, N., et al. (2015). Neural mechanisms of brain plasticity with complex cognitive training in healthy seniors. *Cereb Cortex, 25*(2), 396-405, doi:10.1093/cercor/bht234.

Chapman, S. B., Spence, J. S., Aslan, S., & Keebler, M. W. (2017). Enhancing innovation and underlying neural mechanisms via cognitive training in healthy older adults. *Front Aging Neurosci, 9*, 314, doi:10.3389/fnagi.2017.00314.

Clark, C. M., Lawlor-Savage, L., & Goghari, V. M. (2017). Functional brain activation associated with working memory training and transfer. *Behav Brain Res, 334*, 34-49, doi:10.1016/j.bbr.2017.07.030.

Colom, R., Hua, X., Martinez, K., Burgaleta, M., Roman, F. J., Gunter, J. L., et al. (2016a). Brain structural changes following adaptive cognitive training assessed by tensor-based morphometry (tbm). *Neuropsychologia, 91*, 77-85, doi:10.1016/j.neuropsychologia.2016.07.034.

Colom, R., Martínez, K., Burgaleta, M., Román, F. J., García-García, D., Gunter, J. L., et al. (2016b). Gray matter volumetric changes with a challenging adaptive cognitive training program based on the dual n-back task. *Personality and Individual Differences, 98*, 127-132.

Eack, S. M., Hogarty, G. E., Cho, R. Y., Prasad, K. M., Greenwald, D. P., Hogarty, S. S., et al. (2010). Neuroprotective effects of cognitive enhancement therapy against gray matter loss in early schizophrenia: Results from a 2-year randomized controlled trial. *Arch Gen Psychiatry, 67*(7), 674-682, doi:10.1001/archgenpsychiatry.2010.63.

Eack, S. M., Newhill, C. E., & Keshavan, M. S. (2016). Cognitive enhancement therapy improves resting-state functional connectivity in early course schizophrenia. *J Soc Social Work Res, 7*(2), 211-230, doi:10.1086/686538.

Engvig, A., Fjell, A. M., Westlye, L. T., Moberget, T., Sundseth, O., Larsen, V. A., et al. (2010). Effects of memory training on cortical thickness in the elderly. *Neuroimage, 52*(4), 1667-1676, doi:10.1016/j.neuroimage.2010.05.041.

Engvig, A., Fjell, A. M., Westlye, L. T., Moberget, T., Sundseth, O., Larsen, V. A., et al. (2012). Memory training impacts short-term changes in aging white matter: A longitudinal diffusion tensor imaging study. *Hum Brain Mapp, 33*(10), 2390-2406, doi:10.1002/hbm.21370.

Engvig, A., Fjell, A. M., Westlye, L. T., Skaane, N. V., Dale, A. M., Holland, D., et al. (2014). Effects of cognitive training on gray matter volumes in memory clinic patients with subjective memory impairment. *J Alzheimers Dis, 41*(3), 779-791, doi:10.3233/JAD-131889.

Ernst, A., Sourty, M., Roquet, D., Noblet, V., Gounot, D., Blanc, F., et al. (2018). Benefits from an autobiographical memory facilitation programme in relapsing-remitting multiple sclerosis patients: A clinical and neuroimaging study. *Neuropsychol Rehabil, 28*(7), 1110-1130, doi:10.1080/09602011.2016.1240697.

Fan, F., Zou, Y., Tan, Y., Hong, L. E., & Tan, S. (2017). Computerized cognitive remediation therapy effects on resting state brain activity and cognition in schizophrenia. *Sci Rep, 7*(1), 4758, doi:10.1038/s41598-017-04829-9.

Gaab, N., Gaser, C., & Schlaug, G. (2006). Improvement-related functional plasticity following pitch memory training. *Neuroimage, 31*(1), 255-263, doi:10.1016/j.neuroimage.2005.11.046.

Ghio, M., Locatelli, M., Tettamanti, A., Perani, D., Gatti, R., & Tettamanti, M. (2018). Cognitive training with action-related verbs induces neural plasticity in the action representation system as assessed by gray matter brain morphometry. *Neuropsychologia, 114*, 186-194, doi:10.1016/j.neuropsychologia.2018.04.036.

Hampstead, B. M., Sathian, K., Phillips, P. A., Amaraneni, A., Delaune, W. R., & Stringer, A. Y. (2012a). Mnemonic strategy training improves memory for object location associations in both healthy elderly and patients with amnestic mild cognitive impairment: A randomized, single-blind study. *Neuropsychology, 26*(3), 385-399, doi:10.1037/a0027545.

Hampstead, B. M., Stringer, A. Y., Stilla, R. F., Giddens, M., & Sathian, K. (2012b). Mnemonic strategy training partially restores hippocampal activity in patients with mild cognitive impairment. *Hippocampus, 22*(8), 1652-1658, doi:10.1002/hipo.22006.

Han, K., Chapman, S. B., & Krawczyk, D. C. (2018a). Neuroplasticity of cognitive control networks following cognitive training for chronic traumatic brain injury. *Neuroimage Clin, 18*, 262-278, doi:10.1016/j.nicl.2018.01.030.

Han, K., Davis, R. A., Chapman, S. B., & Krawczyk, D. C. (2017). Strategy-based reasoning training modulates cortical thickness and resting-state functional connectivity in adults with chronic traumatic brain injury. *Brain Behav, 7*(5), e00687, doi:10.1002/brb3.687.

Han, K., Martinez, D., Chapman, S. B., & Krawczyk, D. C. (2018b). Neural correlates of reduced depressive symptoms following cognitive training for chronic traumatic brain injury. *Hum Brain Mapp, 39*(7), 2955-2971, doi:10.1002/hbm.24052.

Heinzel, S., Rimpel, J., Stelzel, C., & Rapp, M. A. (2017). Transfer effects to a multimodal dual-task after working memory training and associated neural correlates in older adults - a pilot study. *Front Hum Neurosci, 11*, 85, doi:10.3389/fnhum.2017.00085.

Hooker, C. I., Bruce, L., Fisher, M., Verosky, S. C., Miyakawa, A., D'Esposito, M., et al. (2013). The influence of combined cognitive plus social-cognitive training on amygdala response during face emotion recognition in schizophrenia. *Psychiatry Res, 213*(2), 99-107, doi:10.1016/j.pscychresns.2013.04.001.

Hooker, C. I., Bruce, L., Fisher, M., Verosky, S. C., Miyakawa, A., & Vinogradov, S. (2012). Neural activity during emotion recognition after combined cognitive plus social cognitive training in schizophrenia. *Schizophr Res, 139*(1-3), 53-59, doi:10.1016/j.schres.2012.05.009.

Hotting, K., Holzschneider, K., Stenzel, A., Wolbers, T., & Roder, B. (2013). Effects of a cognitive training on spatial learning and associated functional brain activations. *BMC Neurosci, 14*, 73, doi:10.1186/1471-2202-14-73.

Hu, M., Wang, X., Zhang, W., Hu, X., & Chen, A. (2017). Neural interactions mediating conflict control and its training-induced plasticity. *Neuroimage, 163*, 390-397, doi:10.1016/j.neuroimage.2017.07.039.

Jiang, L., Cao, X., Li, T., Tang, Y., Li, W., Wang, J., et al. (2016). Cortical thickness changes correlate with cognition changes after cognitive training: Evidence from a chinese community study. *Front Aging Neurosci, 8*, 118, doi:10.3389/fnagi.2016.00118.

Jolles, D. D., van Buchem, M. A., Crone, E. A., & Rombouts, S. A. (2013). Functional brain connectivity at rest changes after working memory training. *Hum Brain Mapp, 34*(2), 396-406, doi:10.1002/hbm.21444.

Kable, J. W., Caulfield, M. K., Falcone, M., McConnell, M., Bernardo, L., Parthasarathi, T., et al. (2017). No effect of commercial cognitive training on brain activity, choice behavior, or cognitive performance. *J Neurosci, 37*(31), 7390-7402, doi:10.1523/JNEUROSCI.2832-16.2017.

Keshavan, M. S., Eack, S. M., Prasad, K. M., Haller, C. S., & Cho, R. Y. (2017). Longitudinal functional brain imaging study in early course schizophrenia before and after cognitive enhancement therapy. *Neuroimage, 151*, 55-64, doi:10.1016/j.neuroimage.2016.11.060.

Kim, G. H., Jeon, S., Im, K., Kwon, H., Lee, B. H., Kim, G. Y., et al. (2015). Structural brain changes after traditional and robot-assisted multi-domain cognitive training in community-dwelling healthy elderly. *PLoS One, 10*(4), e0123251, doi:10.1371/journal.pone.0123251.

Kirchhoff, B. A., Anderson, B. A., Smith, S. E., Barch, D. M., & Jacoby, L. L. (2012). Cognitive training-related changes in hippocampal activity associated with recollection in older adults. *Neuroimage, 62*(3), 1956-1964, doi:10.1016/j.neuroimage.2012.06.017.

Kuhn, S., Schmiedek, F., Noack, H., Wenger, E., Bodammer, N. C., Lindenberger, U., et al. (2013). The dynamics of change in striatal activity following updating training. *Hum Brain Mapp, 34*(7), 1530-1541, doi:10.1002/hbm.22007.

Lee, H., Voss, M. W., Prakash, R. S., Boot, W. R., Vo, L. T., Basak, C., et al. (2012). Videogame training strategy-induced change in brain function during a complex visuomotor task. *Behav Brain Res, 232*(2), 348-357, doi:10.1016/j.bbr.2012.03.043.

Lin, Z. C., Tao, J., Gao, Y. L., Yin, D. Z., Chen, A. Z., & Chen, L. D. (2014). Analysis of central mechanism of cognitive training on cognitive impairment after stroke: Resting-state functional magnetic resonance imaging study. *J Int Med Res, 42*(3), 659-668, doi:10.1177/0300060513505809.

Lorenz, R. C., Gleich, T., Gallinat, J., & Kuhn, S. (2015). Video game training and the reward system. *Front Hum Neurosci, 9*, 40, doi:10.3389/fnhum.2015.00040.

Metzler-Baddeley, C., Caeyenberghs, K., Foley, S., & Jones, D. K. (2016a). Longitudinal data on cortical thickness before and after working memory training. *Data Brief, 7*, 1143-1147, doi:10.1016/j.dib.2016.03.090.

Metzler-Baddeley, C., Caeyenberghs, K., Foley, S., & Jones, D. K. (2016b). Task complexity and location specific changes of cortical thickness in executive and salience networks after working memory training. *Neuroimage, 130*, 48-62, doi:10.1016/j.neuroimage.2016.01.007.

Metzler-Baddeley, C., Foley, S., de Santis, S., Charron, C., Hampshire, A., Caeyenberghs, K., et al. (2017). Dynamics of white matter plasticity underlying working memory training: Multimodal evidence from diffusion mri and relaxometry. *J Cogn Neurosci, 29*(9), 1509-1520, doi:10.1162/jocn_a_01127.

Miotto, E. C., Balardin, J. B., Savage, C. R., Martin Mda, G., Batistuzzo, M. C., Amaro Junior, E., et al. (2014). Brain regions supporting verbal memory improvement in healthy older subjects. *Arq Neuropsiquiatr, 72*(9), 663-670, doi:10.1590/0004-282x20140120.

Miotto, E. C., Savage, C. R., Evans, J. J., Wilson, B. A., Martin, M. G., Balardin, J. B., et al. (2013). Semantic strategy training increases memory performance and brain activity in patients with prefrontal cortex lesions. *Clin Neurol Neurosurg, 115*(3), 309-316, doi:10.1016/j.clineuro.2012.05.024.

Miotto, E. C., Savage, C. R., Evans, J. J., Wilson, B. A., Martins, M. G., Iaki, S., et al. (2006). Bilateral activation of the prefrontal cortex after strategic semantic cognitive training. *Hum Brain Mapp, 27*(4), 288-295, doi:10.1002/hbm.20184.

Miro-Padilla, A., Bueicheku, E., Ventura-Campos, N., Flores-Compan, M. J., Parcet, M. A., & Avila, C. (2019). Long-term brain effects of n-back training: An fmri study. *Brain Imaging Behav, 13*(4), 1115-1127, doi:10.1007/s11682-018-9925-x.

Motes, M. A., Yezhuvath, U. S., Aslan, S., Spence, J. S., Rypma, B., & Chapman, S. B. (2018). Higher-order cognitive training effects on processing speed-related neural activity: A randomized trial. *Neurobiol Aging, 62*, 72-81, doi:10.1016/j.neurobiolaging.2017.10.003.

Mozolic, J. L., Hayasaka, S., & Laurienti, P. J. (2010). A cognitive training intervention increases resting cerebral blood flow in healthy older adults. *Front Hum Neurosci, 4*, 16, doi:10.3389/neuro.09.016.2010.

Murphy, S. E., O'Donoghue, M. C., Blackwell, S. E., Nobre, A. C., Browning, M., & Holmes, E. A. (2017). Increased rostral anterior cingulate activity following positive mental imagery training in healthy older adults. *Soc Cogn Affect Neurosci, 12*(12), 1950-1958, doi:10.1093/scan/nsx120.

Nozawa, T., Taki, Y., Kanno, A., Akimoto, Y., Ihara, M., Yokoyama, R., et al. (2015). Effects of different types of cognitive training on cognitive function, brain structure, and driving safety in senior daily drivers: A pilot study. *Behav Neurol, 2015*, 525901, doi:10.1155/2015/525901.

Osaka, M., Otsuka, Y., & Osaka, N. (2012). Verbal to visual code switching improves working memory in older adults: An fmri study. *Front Hum Neurosci, 6*, 24, doi:10.3389/fnhum.2012.00024.

Ramsay, I. S., Fryer, S., Boos, A., Roach, B. J., Fisher, M., Loewy, R., et al. (2018). Response to targeted cognitive training correlates with change in thalamic volume in a randomized trial for early schizophrenia. *Neuropsychopharmacology, 43*(3), 590-597, doi:10.1038/npp.2017.213.

Raz, N., Schmiedek, F., Rodrigue, K. M., Kennedy, K. M., Lindenberger, U., & Lovden, M. (2013). Differential brain shrinkage over 6 months shows limited association with cognitive practice. *Brain Cogn, 82*(2), 171-180, doi:10.1016/j.bandc.2013.04.002.

Roman, F. J., Iturria-Medina, Y., Martinez, K., Karama, S., Burgaleta, M., Evans, A. C., et al. (2017). Enhanced structural connectivity within a brain sub-network supporting working memory and engagement processes after cognitive training. *Neurobiol Learn Mem, 141*, 33-43, doi:10.1016/j.nlm.2017.03.010.

Roman, F. J., Lewis, L. B., Chen, C. H., Karama, S., Burgaleta, M., Martinez, K., et al. (2016). Gray matter responsiveness to adaptive working memory training: A surface-based morphometry study. *Brain Struct Funct, 221*(9), 4369-4382, doi:10.1007/s00429-015-1168-7.

Schneiders, J. A., Opitz, B., Krick, C. M., & Mecklinger, A. (2011). Separating intra-modal and across-modal training effects in visual working memory: An fmri investigation. *Cereb Cortex, 21*(11), 2555-2564, doi:10.1093/cercor/bhr037.

Schneiders, J. A., Opitz, B., Tang, H., Deng, Y., Xie, C., Li, H., et al. (2012). The impact of auditory working memory training on the fronto-parietal working memory network. *Front Hum Neurosci, 6*, 173, doi:10.3389/fnhum.2012.00173.

Schweizer, S., Grahn, J., Hampshire, A., Mobbs, D., & Dalgleish, T. (2013). Training the emotional brain: Improving affective control through emotional working memory training. *J Neurosci, 33*(12), 5301-5311, doi:10.1523/JNEUROSCI.2593-12.2013.

Subramaniam, K., Luks, T. L., Fisher, M., Simpson, G. V., Nagarajan, S., & Vinogradov, S. (2012). Computerized cognitive training restores neural activity within the reality monitoring network in schizophrenia. *Neuron, 73*(4), 842-853, doi:10.1016/j.neuron.2011.12.024.

Subramaniam, K., Luks, T. L., Garrett, C., Chung, C., Fisher, M., Nagarajan, S., et al. (2014). Intensive cognitive training in schizophrenia enhances working memory and associated prefrontal cortical efficiency in a manner that drives long-term functional gains. *Neuroimage, 99*, 281-292, doi:10.1016/j.neuroimage.2014.05.057.

Sun, J., Chen, Q., Zhang, Q., Li, Y., Li, H., Wei, D., et al. (2016). Training your brain to be more creative: Brain functional and structural changes induced by divergent thinking training. *Hum Brain Mapp, 37*(10), 3375-3387, doi:10.1002/hbm.23246.

Takeuchi, H., Nagase, T., Taki, Y., Sassa, Y., Hashizume, H., Nouchi, R., et al. (2016). Effects of fast simple numerical calculation training on neural systems. *Neural Plast, 2016*, 5940634, doi:10.1155/2016/5940634.

Takeuchi, H., Taki, Y., Hashizume, H., Sassa, Y., Nagase, T., Nouchi, R., et al. (2011a). Effects of training of processing speed on neural systems. *J Neurosci, 31*(34), 12139-12148, doi:10.1523/JNEUROSCI.2948-11.2011.

Takeuchi, H., Taki, Y., Nouchi, R., Hashizume, H., Sekiguchi, A., Kotozaki, Y., et al. (2013). Effects of working memory training on functional connectivity and cerebral blood flow during rest. *Cortex, 49*(8), 2106-2125, doi:10.1016/j.cortex.2012.09.007.

Takeuchi, H., Taki, Y., Nouchi, R., Hashizume, H., Sekiguchi, A., Kotozaki, Y., et al. (2014). Effects of multitasking-training on gray matter structure and resting state neural mechanisms. *Hum Brain Mapp, 35*(8), 3646-3660, doi:10.1002/hbm.22427.

Takeuchi, H., Taki, Y., Nouchi, R., Sekiguchi, A., Kotozaki, Y., Nakagawa, S., et al. (2017). Neural plasticity in amplitude of low frequency fluctuation, cortical hub construction, regional homogeneity resulting from working memory training. *Sci Rep, 7*(1), 1470, doi:10.1038/s41598-017-01460-6.

Takeuchi, H., Taki, Y., Sassa, Y., Hashizume, H., Sekiguchi, A., Fukushima, A., et al. (2011b). Working memory training using mental calculation impacts regional gray matter of the frontal and parietal regions. *PLoS One, 6*(8), e23175, doi:10.1371/journal.pone.0023175.

Thompson, T. W., Waskom, M. L., & Gabrieli, J. D. (2016). Intensive working memory training produces functional changes in large-scale frontoparietal networks. *J Cogn Neurosci, 28*(4), 575-588, doi:10.1162/jocn_a_00916.

Train the Brain Consortium (2017). Randomized trial on the effects of a combined physical/cognitive training in aged mci subjects: The train the brain study. *Sci Rep, 7*, 39471, doi:10.1038/srep39471.

Vartanian, O., Jobidon, M. E., Bouak, F., Nakashima, A., Smith, I., Lam, Q., et al. (2013). Working memory training is associated with lower prefrontal cortex activation in a divergent thinking task. *Neuroscience, 236*, 186-194, doi:10.1016/j.neuroscience.2012.12.060.

Venza, E. E., Chapman, S. B., Aslan, S., Zientz, J. E., Tyler, D. L., & Spence, J. S. (2016). Enhancing executive function and neural health in bipolar disorder through reasoning training. *Front Psychol, 7*, 1676, doi:10.3389/fpsyg.2016.01676.

Wei, D., Yang, J., Li, W., Wang, K., Zhang, Q., & Qiu, J. (2014). Increased resting functional connectivity of the medial prefrontal cortex in creativity by means of cognitive stimulation. *Cortex, 51*, 92-102, doi:10.1016/j.cortex.2013.09.004.

Yin, S., Zhu, X., Li, R., Niu, Y., Wang, B., Zheng, Z., et al. (2014). Intervention-induced enhancement in intrinsic brain activity in healthy older adults. *Sci Rep, 4*, 7309, doi:10.1038/srep07309.

Zheng, Z., Zhu, X., Yin, S., Wang, B., Niu, Y., Huang, X., et al. (2015). Combined cognitive-psychological-physical intervention induces reorganization of intrinsic functional brain architecture in older adults. *Neural Plast, 2015*, 713104, doi:10.1155/2015/713104.
